# IMRT sparing of normal tissues in locoregional treatment of breast cancer

**DOI:** 10.1186/1748-717X-9-161

**Published:** 2014-07-22

**Authors:** Jean-Michel Caudrelier, Joanne Meng, Bernd Esche, Laval Grimard, Terrence Ruddy, Kayvan Amjadi

**Affiliations:** 1 Department of Radiation Medicine, The Ottawa Hospital Cancer Centre, 501, Smyth Rd, Ottawa, ON K1H8L6, Canada; 2 Division of Cardiology and Nuclear Cardiology, Ottawa Heart institute, Ottawa, Canada; 3 Division of Internal Medicine/Respirology, Ottawa Hospital, Ottawa, Canada; 4 Ottawa Hospital Research Institute, Ottawa, Canada

**Keywords:** Breast IMRT, Tomotherapy, Cardiac toxicity, Lung toxicity, Clinical study

## Abstract

**Purpose:**

This clinical study was designed to prospectively evaluate the acute and moderately-late cardiac and lung toxicities of intensity modulated radiation therapy delivered by helical tomotherapy (IMRT-HT) for locoregional breast radiation treatment including the internal mammary nodes (IMN).

**Material/methods:**

30 patients with stage III breast cancers have been accrued in this study. All patients received adjuvant chemotherapy. Target volumes were defined as follows: the PTV included breast/chest wall, axillary level II, III, infra/supraclavicular, IM nodes CTVs plus 3 mm margins. The heart with subunits and the lungs were defined as critical organs. Dose to PTV was 50 Gy in 25 fractions. Acute toxicities were assessed every week and 2 weeks post treatment using the CTCAE v3.0.scale. The moderately-late toxicities were assessed clinically plus by cardiac myoview perfusion tests scheduled at baseline, 3 and 12-month follow-up, as well a CT chest at the 6 month follow-up. The data analysis is descriptive.

**Results:**

All participants completed the 5-week course of radiation without interruption. Skin erythema was modest and mainly grade 1–2 between the 3rd and the 5th week of radiation treatment. Only 4/30 patients experienced grade 3 skin reactions, mostly seen 2 weeks post radiation. Only 5 patients demonstrated grade 1 or 2 dyspnea, but 3 of them already had symptoms pre-radiation treatment. With a median follow-up of 58 (24–76) months, there have been infrequent moderately-late side effects. Most were grade 1 and were sometimes present at the baseline assessment. Cardiac myoview tests done at baseline and 1-year follow-up for 15 out of 18 left sided breast cancers did not show any abnormalities related to radiation. The 6-month follow-up chest CT-scans done for 25 out of 30 patients showed minimal anterior lung fibrosis for 7 patients and were completely normal for the other 18. No locoregional recurrence has been recorded and the 5-year survival is 78% (95% CI: 70-97%).

**Conclusion:**

IMRT-HT for locoregional breast radiation is very well tolerated with minimal acute or moderately-late side effects. Cardiac and respiratory tests did not show any strong evidence of significant treatment related abnormalities.

**Trial registration:**

clinicaltrials.gov: http://NCT00508352.

## Introduction

To irradiate the breast/chest wall and nodal areas, while minimizing the volume of normal tissue within the planning target volume (PTV) can be challenging, in particular if the internal mammary nodes (IMN) are included in the clinical target volume (CTV). Previous clinical trials and meta-analyses have shown that the benefits of locoregional radiation may have been overshadowed by the increased risk of mortality, mainly related to cardiac injury [1-3]. There is evidence that patients treated more recently with updated techniques have no or a lower risk of increased late cardiac mortality [4-6]. However, these patients may still have risks of cardiac morbidity that may also be increased by anthracycline-based chemotherapy regimens potentially combined with trastuzumab [7-10]. As a result, and as the risk/benefit ratio for locoregional radiation is tight, there is significant variation between centres in the use of locoregional radiation, especially when the IMN is included in the target volume [11]. Two clinical trials (The Canadian NCIC MA20 and the EORTC22922–10925) have evaluated the benefits of locoregional radiation, with inclusion of the IMN. Both studies have been completed and the results have been reported in abstract form. Both showed decreasing risks of locoregional recurrence and of metastasis in the group of women treated by locoregional radiation [12,13]. Thus, there is some evidence suggesting that a group of women with breast cancer may benefit from IMN radiation.

Various radiation techniques have been used to encompass the breast/chest wall and nodes including the IMN in order to reduce the dose to the normal tissues including the heart. In recent years, several dosimetry papers have evaluated the benefits of intensity modulated radiotherapy (IMRT) against more conventional 3D techniques [14-23]. They suggest that IMRT results in similar target volume coverage, better target volume conformality and reduced volume of normal tissues irradiated to high dose. Our group published a dosimetric study comparing IMRT-HT versus 3D conventional technique and found similar results in favour of IMRT [23]. Following this dosimetric study, a pilot clinical study was implemented to evaluate the clinical outcomes of patients treated with IMRT-HT for locoregional breast radiation, including the IMN. The eligible women included were those with stage III breast cancer considered at high risk of locoregional recurrence. The goal of this study was to evaluate the acute plus the moderately-late toxicities, with a focus on the heart and lungs. We now report the results, after a minimum follow-up of 47 months in the group of patients still alive.

## Patients and methods

Thirty women with stage III breast cancer, median age of 52 years [33–72], have been accrued in this prospective pilot study between January 2007 and November 2009. Women were eligible if there were any of the following high-risk pathological features present at time of surgery: any pT and pN2, or pT3-4 and pN0-1 with a central/medial location of the tumour. Of the 30 patients, 27 were eventually staged pN2 or pN3 and the last 3 patients who were pN1 had a primary tumour staged pT3. Mastectomy was performed on 20 patients and breast conserving surgery on 10. Eighteen patients had left sided and 12 patients had right-sided breast cancer. All patients received adjuvant chemotherapy, which was completed before starting adjuvant radiation. Patients with Her2neu positive status who were candidates for trastuzamab were not included to avoid confounding factors in evaluation of cardiac toxicity. The main chemotherapy regimens were FEC100-Docetaxel (17 patients) and AC-Paclitaxel (10 patients).

Target volumes were defined on 3 mm CT-scan slices with IV contrast. PTV included the breast/chest wall, axillary level II, III, infra/supraclavicular, and IM nodal CTVs plus a 3 mm margin. A total of 4 radiation oncologists accrued patients and defined the target volumes, using an in-house breast cancer/nodal atlas based on some anatomy books and based on anatomy of previous patients treated. In our center, since 2003 and on yearly basis, an average number of 250 locoregional breast 3D conformal radiation planning, with targeting of nodal areas, is done. The heart (with right and left ventricles and coronary arteries) plus the lungs, oesophagus and thyroid were defined as normal tissue. Dose prescribed to the Planning Target Volume (PTV) was 50 Gy in 25 fractions. Dose constraints on PTV and on normal tissue were defined at time of the dosimetric study and have been previously published [22]. The goal was to deliver 50 Gy to at least 90% of the PTV.

### Clinical outcomes follow-up

A radiation oncologist and/or a clinical research assistant assessed the acute toxicity every week for the duration of treatment and 2 weeks post treatment, using the CTCAE v3.0.scale. Follow-ups were scheduled at 3 months post radiation and then every 6 months thereafter.

Smoking history, cardiac and lung comorbidities were collected. Eleven patients had a smoking history with tobacco consumption from 3 to 45 pack-years, 7 of them having a tobacco consumption at least of 10 pack-years. The prior cardiac history was significant for: 1 patient with valve surgery, 1 patient with bypass surgery, 1 patient with treated arrhythmia and high blood pressure (HBP), plus 5 patients with treated HBP. The respiratory history was significant for: 1 patient with treated chronic obstructive pulmonary disease (the heaviest smoker), and 4 patients with treated asthma.

Cardiac function and radiation effects were evaluated using persantine myoview perfusion and left ventricular function test performed at baseline, 3 and 12-month follow-up. Images for tomographic myocardial perfusion were acquired following persantine stress (50 mg IV) and at rest and by using IV Technetium-99 m tetrofosmin (99mTc) (334 MBq at rest and 1070 MBq at peak stress). Both the myocardial perfusion plus the left ventricular function at rest and post stress were evaluated. The myoview tests were initially done with 99mTc, but following the radioactive metabolite crisis and shortage of 99mTc, follow-up myoview tests were done with Thallium-201 chloride (Tl-201) administered intravenously at peak vasodilation (130 MBq). Cardiac function interpretation combined rating ECG, left ventricular function and myocardial perfusion as normal or not. A global score of zero (if no abnormalities at all) or 1 (if at least 1 abnormality reported) was attributed to each patient. When the global score was 1, tests were reviewed in detail to precisely define the abnormalities reported.

The evaluation of pulmonary toxicity was mainly clinical but a CT scan of the chest was scheduled at the 6-month follow-up to evaluate radiologic fibrosis.

### Statistics

The main objective was to assess the feasibility of IMRT locoregional breast radiation by evaluating the clinical outcomes (radiation side effects). The precision with which the incidence of grade 3 adverse events can be estimated with n = 30 was measured by the 95% confidence interval around the proportion of adverse events, applying a normal approximation to the confidence interval calculation. Even at the maximum of a 50% proportion of adverse events, the confidence interval around the estimate of the proportion of adverse events equals to 33.7%, which provides clinically meaningful estimates. Most of the results are presented in a descriptive way. The survival curve has been estimated by the Kaplan Meier method. Comparison of means, if indicated, was done by a sample *t*-test. Statistical tests were done using XLSTAT 2012.5.02.

This clinical trial was approved by the ethics committee of the Ottawa Hospital Research Institute, patients signed informed consent and the study was registered in the clinicaltrials.gov database with the following identifier: NCT00508352.

## Results

In Table 1, a summary of dosimetric metrics is reported. The doses to the PTV, the lungs, heart and contralateral breast were within the range of what is expected with the IMRT rotational beam technique. We note that for the heart and the coronary arteries, the mean dose, median dose and V25 were globally not different between patients treated to the right or the left side. Only the heart V25 was slightly and significantly higher for the left side.

**Table 1:** Metrics recorded from DVHs

**Treatment planning metrics**		
**PTV**		
V47.5	97.6% ± 1.5%	
V53.5	5.9% ± 6.8%	
**Ipsilateral lung**		
Mean dose	10.2Gy ± 1.1Gy	
V5	49.8% ± 10.4%	
V20	16% ± 3.4%	
**Controlateral lung**		
Mean dose	5.3Gy ± 0.6Gy	
V5	43.7% ± 8.9%	
V20	0.5% ± 1.2%	
**Controlateral breast**		
Mean dose	4.0Gy ± 0.8Gy	
V5	30.3% ± 15.7%	
V10	2.3% ± 4.1%	
**Heart**	**Right side**	**Left side**
Mean dose	7.8Gy ± 1.1Gy	8.0Gy ± 1.2Gy
V25	1.9% ± 1.4%*	3.7% ± 1.9%*
**LAD artery**	**Right side**	**Left side**
Dose max	33.3Gy ± 13.3Gy	26.7Gy ± 15.7Gy
Median dose	13.3Gy ± 5.6Gy	10.8Gy ± 7.8Gy
**RCA artery**	**Right side**	**Left side**
Dose max	30.3Gy ± 8.6Gy	27.0Gy ± 12.4Gy
Median dose	14.0Gy ± 4.9Gy	12.4Gy ± 5.7Gy
**LCx artery**	**Right side**	**Left side**
Dose max	8.6Gy ± 4.4Gy	8.8Gy ± 3.2Gy
Median dose	4.4Gy ± 1.5Gy	4.5Gy ± 1.7Gy

Vx = Volume of target/normal tissue receiving at least x Gy. Values reported are average ± standard deviation of metrics recorded.

*LAD*: left anterior descending artery, *RCA*: right coronary artery, *LCx*: left circumflex artery.

*Comparison of means significantly different between the right and left side treated. *t*-test, *p = 0.01.*

### Acute toxicity

All patients completed the 5-week course of radiation without interruption. Skin erythema was modest and mainly grade 1 during the 3rd and 4th week (22/30 and 24/30 patients, respectively), and grade 2 during the 5th week (22/30 patients). Only 4/30 patients experienced grade 3 (any moist desquamation area) erythema, mostly recorded 2 weeks post radiation. Only 5 patients demonstrated grade 1 or 2 dyspnea but 3 of them already had symptoms pre-irradiation at baseline assessment. One patient had developed acute respiratory distress syndrome pre-irradiation during delivery of adjuvant taxane-based chemotherapy and was managed in the intensive care unit. A second patient developed bronchiolitis obliterans post taxane-based chemotherapy that merited an evaluation by a respirologist. Neither of these women developed any acute respiratory symptoms during radiation treatment. Eleven patients showed grade 1 temporary dysphagia from esophagitis.

### Moderately-late effects

The median follow-up of the whole group, including the deceased patients, is 58 (24–76) months. The evolution of the major moderately-late side effects is summarized in Table 2. Skin reactions, breast/chest-wall pain, respiratory and sensori-motor symptoms have been infrequent, and mostly grade 1. They were sometimes already present at baseline assessment. Six patients underwent breast reconstruction post mastectomy and none developed any significant complications, including no arm lymphedema recorded from physical examination or from arm measurements done by a nurse. One patient was diagnosed with acute leukemia 24 months post radiation treatment and is currently in complete remission post treatment. Her adjuvant chemotherapy regimen was FEC100-Docetaxel. One patient was diagnosed with a small cell lung cancer on the radiated side 30 months post radiation treatment. This patient had other lung cancer risk factors. The primary tumour was localized in an area that was covered by 40 to 50 Gy. It is possible that this small cell lung cancer was radiation induced. However, this causality is not specific to the IMRT technique as a tangential radiation beams technique would have covered the same area with the 40 to 50 Gy isodoses.

**Table 2:** The most frequent moderately-late side effects recorded

**Moderately-late side effects**								
**Adverse events**	**Grade**	**Baseline**	**3 months**	**6 months**	**12 months**	**18 months**	**24 months**	**30 months**
		**30 pts**	**30 pts**	**30 pts**	**29 pts**	**28 pts**	**27 pts**	**26 pts**
**Erythema pigmentation**	**Grade 0**		6	14	18	22	23	22
	**Grade1**		23	16	11	6	4	4
	**Grade2**		1	0	0	0	0	0
**Induration fibrosis**	**Grade 0**		23	23	22	24	23	22
	**Grade 1**		6	6	7	4	4	4
	**Grade 2**		1	1	0	0	0	0
**Telangiectasia**	**Grade 0**		30	30	29	26	23	21
	**Grade 1**		0	0	0	2	4	4
	**Grade 2**		0	0	0	0	0	1
**Breast/chest wall pain**	**Grade 0**	23	13	16	17	18	20	20
	**Grade 1**	6	16	13	12	9	6	6
	**Grade 2**	1	0	1	0	1	1	0
	**Grade 3**	0	1	0	0	0	0	0
**Cough/dyspnea**	**Grade 0**	27	28	28	26	25	26	26
	**Grade 1**	3	2	2	3	2	1	0
**MSK upper limb treated/neurosensitive symptoms**	**Grade 0**	18	24	25	25	25	25	26
	**Grade 1**	10	5	4	4	3	3	1
	**Grade 2**	2	1	1	0	0	0	0

The only grade 2 reported at 30 months is a cosmetic one. Note that the number of grade 1 toxicity is low at 30 months, with significant decreased number over time for pain and skin erythema/pigmentation.

We can note that the number of grade 1 or 2 side effects for cough/dyspnea and neuro-muscular symptoms are not very significantly different between baseline and early follow-up assessments.

MSK: musculoskeletal.

### Myoview scintigraphy tests

Women were scheduled for baseline, 3 and 12-month follow-up scintigraphy but some women declined the initial tests and some declined any follow-up tests, mostly because of the discomfort caused by the persantine injection used to simulate stress. The number of tests done and abnormalities reported are summarized in Table 3. Abnormalities have been recorded for 6 women, 4 radiated on the right side and 2 on the left. For 5 patients, the perfusion abnormalities are not related to the radiation treatment. Three were present at baseline and 2 at follow-up were related to imaging artefacts according to cardiology assessment. Only 1 patient’s abnormalities were possibly related to radiation. This patient had several cardiac comorbidities (arrhythmia, HBP, smoking habits) and her cardiac radiation doses were the following: heart mean dose:7.8Gy, V25:4%; left ventricle median dose:4.4Gy [2.2-28.9], V30:0%; LAD artery median dose:26Gy [3.7-43.5]. Following a gastrointestinal surgical procedure, this patient died from congestive heart failure post atrial flutter.

**Table 3:** Patients with myoview perfusion cardiac tests having at least one abnormal study

**Myoview perfusion cardiac tests**				
	**Baseline**	**3 months F-up**	**12 months F-up**	
**Patient number and side radiated**	**26 tests 99 m Tc: 21 Tl-201: 5**	**21 tests 99 m Tc: 13 Tl-201: 8**	**23 tests 99 m Tc: 9 Tl-201: 14**	**Comments**
1: Right	P	P	P	History of cardiac surgery
2: Right	P	?	0	Artefact
3: Left	P	0	0	Artefact
4: Right	0	P	?	No cardiac event
5: Left	0	?	P*	*positive test 30 months post radiation
6: Right	0	0	P	Artefact

*Note that the patient number 5 had a test positive 30 months post radiation. This patient declined any per protocol follow-up myoview test.

P: perfusion abnormality, 0: no perfusion abnormality, ?: test not done.

### Pulmonary toxicity

As summarized in Table 2, no significant late clinical pulmonary toxicity has been recorded. A maximum number of 3 patients experienced a grade 1 dyspnea or cough from 3 and up to the 12 month follow-up. The 2 patients who needed respirologist evaluation were all seen during or at the end of chemotherapy. At 30 months, all 26 remaining patients had no respiratory symptoms. A 6-month follow-up CT chest has been done for 25 out of 30 patients. For 18 patients, the CT chest was completely normal and for the other 7, the CT chest showed a minimal anterior fibrosis (grade 1 fibrosis).

### Recurrences/survival

No loco-regional recurrence has been diagnosed. Five patients have been diagnosed with distant recurrent disease. One patient developed a contralateral breast cancer with axillary nodal involvement at 11 months post completion of radiation treatment. This was in the context of a BRCA1 gene mutation. The same patient later developed metastatic disease. Four patients have been diagnosed with bone metastasis at least after 6 months of follow-up. Six deaths have been recorded: 4 patients from progression of metastatic breast cancer, 1 patient from a lung cancer and one due to a cardiac event post surgical procedure. The estimated Kaplan Meier survival at 5 years is 78% (CI 95%: 70-97%).

## Discussion

Planning of locoregional breast radiation is challenging, particularly when the IMN is included. IMRT techniques for locoregional breast radiation have been evaluated in multiple dosimetric papers. However, publications of clinical studies of such locoregional breast radiation techniques are still limited. Within the frame of this pilot study involving a limited number of patients and with the dose constraints used for PTV and normal tissue, the acute toxicities were modest. The rate and the degree of erythema were similar to the acute skin reactions reported in one recent clinical trial evaluating intact breast IMRT [24]. The moderately-late skin side effects such as erythema and/or pigmentation were also decreasing over time and only a small number of patients showed any residual skin effects.

### Lung tolerance

Clinically significant symptomatic radiation pneumonitis occurs in approximately 1–5% of patients irradiated for breast cancers [25]. One of the major concerns (explaining the low rate of accrual) with this pilot trial was the possibility of an increased risk of severe subacute respiratory symptoms related to the IMRT rotational beam technique. This technique results in an increase in the volume of lung receiving a low dose of radiation (<10Gy). Severe respiratory toxicity has been reported for mesothelioma patients treated by post operative IMRT when the mean lung dose and V20 and V5 were increased [26]. However in our group of patients, acute or moderately late lung effects were not seen. Most of the respiratory symptoms recorded were already present at baseline and some were partly related to the taxane-based regimen. These results may fit with some published data suggesting that reducing the volume of lung receiving high dose of radiation (≥40Gy) and reducing the mean lung dose decrease the risks of radiation pneumonitis [27]. The CT chest at the 6-month follow-up done for 25 (83%) of the patients was reported normal for 18 (72%) of them. The others showed minimal anterior fibrosis. Thus, we may postulate that locoregional IMRT was very well tolerated regarding pulmonary side effects. It could be noted that the average DVH values recorded for lungs in our clinical study are in the range of values recommended for radiation therapy post pneumonectomy for mesothelioma in the last Quantitative Analysis of Normal Tissue Effects in the Clinic (QUANTEC) review [28].

### Cardiac effects

Cardiac perfusion defects of up to 30% have been seen on follow-up scintigraphy perfusion tests for irradiated left breast cancer patients [29-31]. Myoview tests have been used as a surrogate marker of possible cardiac injury from radiation treatment. Fifteen out of eighteen left sided breast cancer patients had baseline, 3 and 12 month follow-up myoview tests and none of them detected a significant perfusion abnormality. The only patient who developed cardiac problems was diagnosed with an abnormal test at 30 months post radiation treatment. It is unclear if these abnormalities and subsequent cardiac problems were related to previous breast cancer treatments, but we cannot completely exclude radiation treatment causality for this patient. A recent study reported a percent increase of major coronary event by 7.4% for every 1 Gy of mean cardiac dose [10]. For a mean cardiac dose of 8 Gy (the mean cardiac dose recorded in our study for women radiated on the left or right side) the estimated percent increase of major coronary event is 40%. It is unclear if this estimated number from the Darby et al. paper can be used for locoregional breast radiation with IMRT rotational beam technique. Their study reported only the mean cardiac dose, but not the volume of heart receiving above 25 Gy and/or close to the prescription dose. Radiation by a combination of tangent beams treats a larger volume of heart with a dose equal or superior to 25 Gy, compare to IMRT rotational beam technique. On the other hand, IMRT rotational beam technique will radiate a larger volume of heart to a dose less than or equal to 10 Gy, and as a result the mean cardiac dose will be higher compared to the tangential beam radiation technique. We think that when evaluating a locoregional breast radiation planning, the mean cardiac dose is not the only factor to consider and that the V25 and/or V30, V40 are also important metrics. From the perfusion myoview tests results, we also note that a mean cardiac dose around 6–8 Gy seems to be safe for breast cancer women, if we can achieve a V25 below 5% and a V30 below 2.5%. We do not believe that the mean cardiac dose is the only metric to meaningfully compare 3D planning using tangent beams and the IMRT rotational beam technique.

As stated above and due to the shortage of radionuclides, 99mTc was replaced by Tl-201. One may argue that this may have altered the results of the myoview tests. However, the ROBUST study compared the performances of 3 radionuclide myocardial perfusion tracers for clinical practice in a large population of 2560 patients. That study concluded that all tracers performed well in clinical terms, with high sensitivity and specificity for angiographic stenosis and no differences in accuracy between the tracers [32]. Therefore, we may reasonably suggest that the negativity of myoview tests is likely due to the sparing of cardiac structure including the coronary arteries from a high dose of radiation.

## Conclusion

Within the frame of this prospective evaluation done on a limited number of patients, IMRT-HT for locoregional breast radiation has been feasible and very well tolerated with minimal acute or moderately-late side effects. Cardiac and respiratory tests did not show any significant treatment related abnormalities. In our cancer centre, IMRT-HT is now a routine option when a complex locoregional radiation is indicated. Since 2011, we have also have implemented volumetric linac based IMRT.

## Competing interests

None to declare for all authors.

## Authors’ contribution

JMC: conception and design of the study, accrual of patients, analysis and interpretation of data, writing of manuscript. JM: accrual of patients, revision of manuscript. BE: accrual of patients, revision of manuscript. LG: accrual of patients, revision of manuscript. TR: design of the study, interpretation of cardiac data, revision of manuscript. KA: design of the study, revision of manuscript. All authors read and approved the final manuscript.
